# Diethyl (1-hydr­oxy-1,2-diphenyl­ethyl)phospho­nate

**DOI:** 10.1107/S160053680901602X

**Published:** 2009-05-07

**Authors:** Nurcan Acar, M. Nawaz Tahir, Riaz H. Tariq, Hamza Yilmaz

**Affiliations:** a Department of Chemistry, Faculty of Science, University of Ankara, Ankara, Turkey; b Department of Physics, University of Sargodha, Sargodha, Pakistan; c Department of Pharmacy, University of Faisalabad, Faisalabad, Pakistan

## Abstract

In the title compound, C_18_H_23_O_4_P, the dihedral angle between the aromatic ring planes is 69.94 (14)°. Both ethyl side chains are disordered over two sets of sites, with occupancy ratios of 80:20 and 70:30. In the crystal, inversion dimers linked by pairs of O—H⋯O hydrogen bonds occur, leading to *R*_2_^1^(8) loops, and C—H⋯O and weak C—H⋯π inter­actions are also seen.

## Related literature

For related structures, see: Acar *et al.* (2009 ▶); Tahir *et al.* (2007 ▶, 2009*a* ▶,*b* ▶). For the synthesis, see: Texier-Boullet & Lequitte (1986 ▶). For graph-set notation, see: Bernstein *et al.* (1995 ▶).
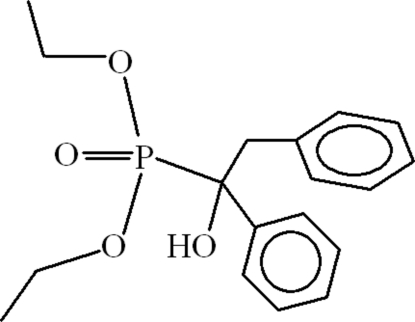


## Experimental

### 

#### Crystal data

C_18_H_23_O_4_P
*M* *_r_* = 334.33
Monoclinic, 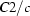

*a* = 23.8545 (11) Å
*b* = 10.6663 (5) Å
*c* = 18.4994 (14) Å
β = 129.451 (2)°
*V* = 3634.6 (4) Å^3^
*Z* = 8
Mo *K*α radiation
μ = 0.17 mm^−1^
*T* = 296 K
0.30 × 0.25 × 0.22 mm

#### Data collection

Bruker Kappa APEXII CCD diffractometer
Absorption correction: multi-scan (*SADABS*; Bruker, 2005 ▶) *T*_min_ = 0.953, *T*_max_ = 0.968
18572 measured reflections
4354 independent reflections
2742 reflections with *I* > 2σ(*I*)
*R*_int_ = 0.031

#### Refinement

*R*[*F* ^2^ > 2σ(*F* ^2^)] = 0.065
*wR*(*F* ^2^) = 0.198
*S* = 1.05
4354 reflections
217 parameters
8 restraints
H atoms treated by a mixture of independent and constrained refinement
Δρ_max_ = 0.73 e Å^−3^
Δρ_min_ = −0.63 e Å^−3^

### 

Data collection: *APEX2* (Bruker, 2007 ▶); cell refinement: *SAINT* (Bruker, 2007 ▶); data reduction: *SAINT*; program(s) used to solve structure: *SHELXS97* (Sheldrick, 2008 ▶); program(s) used to refine structure: *SHELXL97* (Sheldrick, 2008 ▶); molecular graphics: *ORTEP-3* (Farrugia, 1997 ▶) and *PLATON* (Spek, 2009 ▶); software used to prepare material for publication: *WinGX* (Farrugia, 1999 ▶) and *PLATON*.

## Supplementary Material

Crystal structure: contains datablocks global, I. DOI: 10.1107/S160053680901602X/hb2959sup1.cif

Structure factors: contains datablocks I. DOI: 10.1107/S160053680901602X/hb2959Isup2.hkl

Additional supplementary materials:  crystallographic information; 3D view; checkCIF report

## Figures and Tables

**Table 1:** Hydrogen-bond geometry (Å, °)

*D*—H⋯*A*	*D*—H	H⋯*A*	*D*⋯*A*	*D*—H⋯*A*
O1—H1⋯O4^i^	0.86 (4)	1.87 (4)	2.718 (3)	170 (5)
C10—H10⋯O4^i^	0.93	2.54	3.409 (4)	155
C16*A*—H16*B*⋯CgA^ii^	0.97	2.94	3.657 (8)	133

Symmetry codes: (i) 
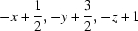
; (ii) 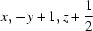. *CgA* is the centroid of the C9–C14 ring.

**Fig. 1.**
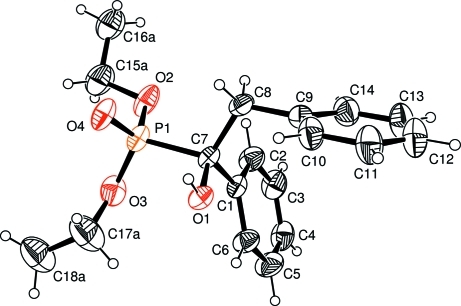
View of (I) showing 30% displacement ellipoids (H-atoms are shown by small spheres of arbitrary radius). The ethyl moieties of smaller occupancy factor are not shown for clarity.

**Fig. 2.**
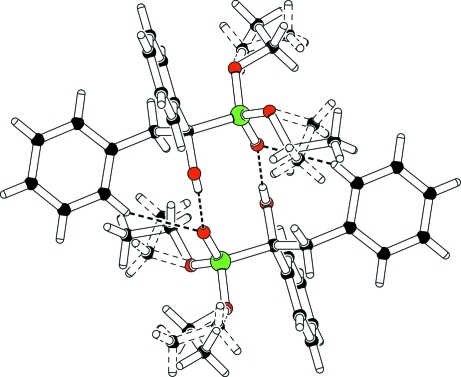
Detail of (I) showing the formation of dimers through hydrogen bonding forming R22(10) motifs.

## supplementary crystallographic information

### Comment

We have reported the preparation and crystal structures of the phosphonate compounds (Acar *et al.*, 2009; Tahir *et al.*, 2007, 2009*a*, 2009*b*). In continuation to the study of phosphonate compounds, we, herein report the preparation and crystal structure of the title compound (I), (Fig. 1).

The crystal structures of (II) Dimethyl (1-hydroxy-1,2-diphenylethyl)phosphonate (Acar *et al.*, 2009) has been reported which differs from (I) due to dimethylphosphonate instead of diethylphosphonate. In the title compound ethyl moieties of diethylphosphonate are disordered over two sites with occupancy ratios of 80:20 and 70:30, respectively. The disorder in diethyl moieties is also present in the reported structure (Tahir *et al.*,  2009*a*). The present compound is basically dimerized forming ring motifs *R*_2_^2^(10) (Bernstein *et al.*, 1995) due to O—H···O type of intermolecular H-bonding (Fig. 2). The title molecule is also stabilized due to intermolecular H-bonding of C—H···O type and C—H···CgA interaction (Table 1), Where CgA is the centroid of the aromatic ring A (C9–C14). Ring motifs of *R*_2_^1^(8) are also present which are formed due to the intermolecular H-bondings (Fig. 2). The dihedral angle between the ring A and the benzene ring B (C1–C6) is 69.94 (14)°.

### Experimental

The title compound was prepared according to method described by Texier-Boullet & Lequitte (1986). In a solution mixture of 2-phenylacetophenone (3.92 g, 20 mmol) and diethylphosphonate (2.76 g, 20 mmol), a mixture of KF (5 g, 86.20 mmol) and commercial Al_2_O_3_ (5 g, 49 mmol) was slowly added at 273 K. The reaction mixture remained at room temperature for two days. The product was extracted twice with CH_3_Cl_2_. The extracted product was recrystallized in a mixture of distalled water and ethyl alcohol to yield colourless prisms of (I).

### Refinement

The coordinates of H-atom of hydroxy group were refined. C-bound H atoms were positioned geometrically, with C-H = 0.93, 0.96 and 0.97 Å for aromatic, methyl and ethylene moieties and constrained to ride on their parent atoms, with U_iso_(H) = xU_eq_(C, O), where x = 1.5 for methyl H, and x = 1.2 for all other H atoms.

### Crystal data

**Table:**

C_18_H_23_O_4_P	*F*(000) = 1424
*M**_r_* = 334.33	*D*_x_ = 1.220 Mg m^−^^3^
Monoclinic, *C*2/*c*	Mo *K*α radiation, λ = 0.71073 Å
Hall symbol: -C 2yc	Cell parameters from 4354 reflections
*a* = 23.8545 (11) Å	θ = 2.2–27.9°
*b* = 10.6663 (5) Å	µ = 0.17 mm^−^^1^
*c* = 18.4994 (14) Å	*T* = 296 K
β = 129.451 (2)°	Prism, colourless
*V* = 3634.6 (4) Å^3^	0.30 × 0.25 × 0.22 mm
*Z* = 8	

### Data collection

**Table:**

Bruker Kappa APEXII CCD diffractometer	4354 independent reflections
Radiation source: fine-focus sealed tube	2742 reflections with *I* > 2σ(*I*)
graphite	*R*_int_ = 0.031
Detector resolution: 7.50 pixels mm^-1^	θ_max_ = 27.9°, θ_min_ = 2.2°
ω scans	*h* = −27→31
Absorption correction: multi-scan (*SADABS*; Bruker, 2005)	*k* = −13→14
*T*_min_ = 0.953, *T*_max_ = 0.968	*l* = −23→24
18572 measured reflections	

### Refinement

**Table:**

Refinement on *F*^2^	Primary atom site location: structure-invariant direct methods
Least-squares matrix: full	Secondary atom site location: difference Fourier map
*R*[*F*^2^ > 2σ(*F*^2^)] = 0.065	Hydrogen site location: inferred from neighbouring sites
*wR*(*F*^2^) = 0.198	H atoms treated by a mixture of independent and constrained refinement
*S* = 1.05	*w* = 1/[σ^2^(*F*_o_^2^) + (0.0851*P*)^2^ + 3.8003*P*] where *P* = (*F*_o_^2^ + 2*F*_c_^2^)/3
4354 reflections	(Δ/σ)_max_ < 0.001
217 parameters	Δρ_max_ = 0.73 e Å^−^^3^
8 restraints	Δρ_min_ = −0.63 e Å^−^^3^

### Special details

**Table:**

Geometry. Bond distances, angles *etc*. have been calculated using the rounded fractional coordinates. All su's are estimated from the variances of the (full) variance-covariance matrix. The cell e.s.d.'s are taken into account in the estimation of distances, angles and torsion angles
Refinement. Refinement of *F*^2^ against ALL reflections. The weighted *R*-factor *wR* and goodness of fit *S* are based on *F*^2^, conventional *R*-factors *R* are based on *F*, with *F* set to zero for negative *F*^2^. The threshold expression of *F*^2^ > σ(*F*^2^) is used only for calculating *R*-factors(gt) *etc*. and is not relevant to the choice of reflections for refinement. *R*-factors based on *F*^2^ are statistically about twice as large as those based on *F*, and *R*- factors based on ALL data will be even larger.

### Fractional atomic coordinates and isotropic or equivalent isotropic displacement parameters (Å^2^)

**Table:**

	*x*	*y*	*z*	*U*_iso_*/*U*_eq_	Occ. (<1)
P1	0.23098 (5)	0.51952 (7)	0.48295 (5)	0.0564 (3)	
O1	0.24033 (11)	0.65326 (19)	0.37591 (14)	0.0534 (7)	
O2	0.17697 (15)	0.4238 (2)	0.47220 (16)	0.0809 (10)	
O3	0.29721 (15)	0.4365 (2)	0.51385 (17)	0.0789 (9)	
O4	0.25082 (14)	0.62304 (19)	0.54704 (14)	0.0684 (8)	
C1	0.17620 (15)	0.4629 (3)	0.30222 (18)	0.0499 (9)	
C2	0.1240 (2)	0.3727 (3)	0.2725 (2)	0.0713 (12)	
C3	0.1151 (2)	0.2728 (4)	0.2178 (3)	0.0802 (14)	
C4	0.1576 (2)	0.2619 (3)	0.1929 (2)	0.0781 (14)	
C5	0.2088 (2)	0.3494 (4)	0.2220 (3)	0.0832 (16)	
C6	0.21863 (19)	0.4501 (3)	0.2765 (2)	0.0656 (12)	
C7	0.18743 (15)	0.5724 (3)	0.36315 (18)	0.0473 (8)	
C8	0.11690 (18)	0.6435 (3)	0.3225 (2)	0.0603 (11)	
C9	0.08167 (17)	0.7045 (3)	0.2292 (2)	0.0604 (11)	
C10	0.1049 (2)	0.8214 (3)	0.2239 (2)	0.0742 (11)	
C11	0.0723 (2)	0.8779 (4)	0.1386 (3)	0.0914 (14)	
C12	0.0159 (3)	0.8215 (4)	0.0578 (3)	0.1001 (16)	
C13	−0.0075 (2)	0.7068 (5)	0.0618 (3)	0.0986 (18)	
C14	0.02499 (19)	0.6485 (4)	0.1464 (3)	0.0802 (14)	
C15A	0.1966 (3)	0.3662 (6)	0.5570 (4)	0.1000 (17)	0.800
C16A	0.1312 (3)	0.3721 (6)	0.5486 (4)	0.1000 (17)	0.800
C17A	0.3652 (4)	0.4948 (10)	0.5515 (7)	0.130 (3)	0.700
C18A	0.4280 (4)	0.4270 (8)	0.6305 (6)	0.130 (3)	0.700
C17B	0.3660 (8)	0.434 (2)	0.6034 (12)	0.130 (3)	0.300
C15B	0.1434 (12)	0.424 (2)	0.5136 (14)	0.1000 (17)	0.200
C16B	0.1959 (13)	0.347 (2)	0.5989 (15)	0.1000 (17)	0.200
C18B	0.3938 (11)	0.497 (3)	0.5609 (19)	0.130 (3)	0.300
H1	0.241 (2)	0.719 (4)	0.403 (3)	0.0799*	
H6	0.25426	0.50943	0.29577	0.0793*	
H8A	0.08260	0.58537	0.31588	0.0722*	
H8B	0.12741	0.70761	0.36680	0.0722*	
H2	0.09468	0.37888	0.28914	0.0847*	
H3	0.07968	0.21271	0.19812	0.0963*	
H4	0.15139	0.19501	0.15622	0.0936*	
H5	0.23811	0.34205	0.20531	0.0991*	
H14	0.00846	0.57023	0.14766	0.0964*	
H15A	0.23620	0.41156	0.61197	0.1200*	0.800
H15B	0.21144	0.27982	0.56195	0.1200*	0.800
H16A	0.11604	0.45783	0.54114	0.1500*	0.800
H16B	0.14165	0.33803	0.60407	0.1500*	0.800
H16C	0.09302	0.32440	0.49513	0.1500*	0.800
H17A	0.36568	0.57932	0.57130	0.1557*	0.700
H17B	0.36904	0.50098	0.50248	0.1557*	0.700
H18A	0.44289	0.46306	0.68793	0.1947*	0.700
H18B	0.46708	0.43278	0.62840	0.1947*	0.700
H18C	0.41540	0.34059	0.62738	0.1947*	0.700
H10	0.14295	0.86168	0.27863	0.0893*	
H11	0.08898	0.95540	0.13628	0.1095*	
H12	−0.00657	0.86058	0.00042	0.1191*	
H13	−0.04578	0.66759	0.00674	0.1182*	
H15C	0.09576	0.38470	0.47291	0.1200*	0.200
H15D	0.13903	0.50761	0.52946	0.1200*	0.200
H16D	0.17588	0.26456	0.59053	0.1500*	0.200
H16E	0.20506	0.38620	0.65210	0.1500*	0.200
H16F	0.24055	0.33917	0.60908	0.1500*	0.200
H17C	0.38530	0.35052	0.62670	0.1557*	0.300
H17D	0.36927	0.48414	0.64973	0.1557*	0.300
H18D	0.39393	0.43929	0.52126	0.1947*	0.300
H18E	0.44239	0.52619	0.60936	0.1947*	0.300
H18F	0.36325	0.56736	0.52431	0.1947*	0.300

### Atomic displacement parameters (Å^2^)

**Table:**

	*U*^11^	*U*^22^	*U*^33^	*U*^12^	*U*^13^	*U*^23^
P1	0.0822 (6)	0.0449 (4)	0.0468 (4)	−0.0038 (4)	0.0432 (4)	−0.0023 (3)
O1	0.0690 (13)	0.0474 (11)	0.0556 (11)	−0.0050 (10)	0.0452 (10)	−0.0051 (9)
O2	0.122 (2)	0.0731 (15)	0.0634 (14)	−0.0310 (14)	0.0663 (15)	−0.0082 (12)
O3	0.0938 (18)	0.0634 (14)	0.0675 (14)	0.0183 (13)	0.0456 (13)	0.0055 (12)
O4	0.1092 (18)	0.0490 (12)	0.0520 (12)	−0.0039 (12)	0.0535 (12)	−0.0053 (9)
C1	0.0603 (16)	0.0497 (16)	0.0407 (13)	0.0008 (13)	0.0326 (13)	−0.0008 (11)
C2	0.078 (2)	0.074 (2)	0.069 (2)	−0.0178 (18)	0.0501 (18)	−0.0185 (17)
C3	0.089 (3)	0.071 (2)	0.068 (2)	−0.025 (2)	0.044 (2)	−0.0217 (18)
C4	0.097 (3)	0.066 (2)	0.058 (2)	0.000 (2)	0.043 (2)	−0.0160 (16)
C5	0.097 (3)	0.090 (3)	0.082 (2)	−0.004 (2)	0.066 (2)	−0.025 (2)
C6	0.077 (2)	0.069 (2)	0.068 (2)	−0.0109 (17)	0.0541 (18)	−0.0174 (16)
C7	0.0609 (16)	0.0482 (15)	0.0432 (13)	−0.0041 (13)	0.0379 (13)	−0.0029 (11)
C8	0.0692 (19)	0.071 (2)	0.0582 (17)	0.0050 (16)	0.0487 (16)	0.0000 (15)
C9	0.0586 (18)	0.071 (2)	0.0551 (17)	0.0118 (15)	0.0378 (15)	0.0033 (15)
C10	0.079 (2)	0.066 (2)	0.0573 (19)	0.0130 (18)	0.0338 (17)	0.0025 (16)
C11	0.101 (3)	0.071 (2)	0.071 (2)	0.013 (2)	0.040 (2)	0.0162 (19)
C12	0.102 (3)	0.099 (3)	0.060 (2)	0.015 (3)	0.033 (2)	0.018 (2)
C13	0.076 (3)	0.118 (4)	0.057 (2)	−0.003 (3)	0.0213 (19)	0.003 (2)
C14	0.066 (2)	0.095 (3)	0.067 (2)	−0.007 (2)	0.0364 (18)	0.004 (2)
C15A	0.125 (3)	0.104 (3)	0.091 (3)	−0.004 (3)	0.078 (3)	0.017 (2)
C16A	0.125 (3)	0.104 (3)	0.091 (3)	−0.004 (3)	0.078 (3)	0.017 (2)
C17A	0.084 (4)	0.133 (4)	0.136 (5)	0.020 (4)	0.053 (4)	0.032 (3)
C18A	0.084 (4)	0.133 (4)	0.136 (5)	0.020 (4)	0.053 (4)	0.032 (3)
C17B	0.084 (4)	0.133 (4)	0.136 (5)	0.020 (4)	0.053 (4)	0.032 (3)
C15B	0.125 (3)	0.104 (3)	0.091 (3)	−0.004 (3)	0.078 (3)	0.017 (2)
C16B	0.125 (3)	0.104 (3)	0.091 (3)	−0.004 (3)	0.078 (3)	0.017 (2)
C18B	0.084 (4)	0.133 (4)	0.136 (5)	0.020 (4)	0.053 (4)	0.032 (3)

### Geometric parameters (Å, °)

**Table:**

P1—O2	1.554 (4)	C4—H4	0.9300
P1—O3	1.568 (4)	C5—H5	0.9300
P1—O4	1.461 (2)	C6—H6	0.9300
P1—C7	1.839 (3)	C8—H8A	0.9700
O1—C7	1.419 (5)	C8—H8B	0.9700
O2—C15A	1.458 (7)	C10—H10	0.9300
O2—C15B	1.42 (3)	C11—H11	0.9300
O3—C17A	1.438 (12)	C12—H12	0.9300
O3—C17B	1.408 (19)	C13—H13	0.9300
O1—H1	0.86 (4)	C14—H14	0.9300
C1—C2	1.382 (6)	C15A—H15A	0.9700
C1—C7	1.524 (4)	C15A—H15B	0.9700
C1—C6	1.372 (6)	C15B—H15C	0.9700
C2—C3	1.390 (6)	C15B—H15D	0.9600
C3—C4	1.359 (8)	C16A—H16A	0.9600
C4—C5	1.348 (7)	C16A—H16C	0.9600
C5—C6	1.387 (6)	C16A—H16B	0.9600
C7—C8	1.537 (6)	C16B—H16E	0.9600
C8—C9	1.505 (4)	C16B—H16D	0.9700
C9—C14	1.378 (5)	C16B—H16F	0.9600
C9—C10	1.393 (5)	C17A—H17A	0.9700
C10—C11	1.378 (5)	C17A—H17B	0.9700
C11—C12	1.360 (6)	C17B—H17C	0.9700
C12—C13	1.366 (8)	C17B—H17D	0.9700
C13—C14	1.377 (6)	C18A—H18A	0.9600
C15A—C16A	1.468 (12)	C18A—H18B	0.9600
C15B—C16B	1.49 (3)	C18A—H18C	0.9600
C17A—C18A	1.456 (14)	C18B—H18D	0.9600
C17B—C18B	1.47 (4)	C18B—H18E	0.9600
C2—H2	0.9300	C18B—H18F	0.9600
C3—H3	0.9300		
			
O2—P1—O3	103.70 (16)	C11—C10—H10	120.00
O2—P1—O4	114.51 (19)	C10—C11—H11	120.00
O2—P1—C7	105.05 (15)	C12—C11—H11	120.00
O3—P1—O4	113.52 (16)	C11—C12—H12	120.00
O3—P1—C7	106.21 (17)	C13—C12—H12	120.00
O4—P1—C7	112.93 (13)	C12—C13—H13	120.00
P1—O2—C15A	117.8 (3)	C14—C13—H13	120.00
P1—O2—C15B	129.6 (9)	C9—C14—H14	120.00
P1—O3—C17A	119.8 (5)	C13—C14—H14	120.00
P1—O3—C17B	126.2 (9)	O2—C15A—H15A	111.00
C7—O1—H1	106 (4)	O2—C15A—H15B	111.00
C2—C1—C6	118.0 (3)	C16A—C15A—H15A	111.00
C2—C1—C7	121.2 (4)	C16A—C15A—H15B	111.00
C6—C1—C7	120.7 (3)	H15A—C15A—H15B	109.00
C1—C2—C3	120.4 (5)	O2—C15B—H15D	112.00
C2—C3—C4	120.7 (4)	C16B—C15B—H15C	111.00
C3—C4—C5	119.2 (4)	O2—C15B—H15C	111.00
C4—C5—C6	121.2 (5)	H15C—C15B—H15D	110.00
C1—C6—C5	120.5 (4)	C16B—C15B—H15D	112.00
P1—C7—O1	102.96 (19)	C15A—C16A—H16B	110.00
P1—C7—C8	110.2 (3)	C15A—C16A—H16A	110.00
O1—C7—C1	108.0 (3)	C15A—C16A—H16C	110.00
P1—C7—C1	110.8 (2)	H16A—C16A—H16B	109.00
C1—C7—C8	113.1 (3)	H16A—C16A—H16C	109.00
O1—C7—C8	111.3 (3)	H16B—C16A—H16C	109.00
C7—C8—C9	114.5 (4)	C15B—C16B—H16E	110.00
C8—C9—C10	120.7 (3)	C15B—C16B—H16F	110.00
C8—C9—C14	121.7 (3)	C15B—C16B—H16D	110.00
C10—C9—C14	117.6 (3)	H16D—C16B—H16E	109.00
C9—C10—C11	120.8 (3)	H16D—C16B—H16F	109.00
C10—C11—C12	120.6 (4)	H16E—C16B—H16F	110.00
C11—C12—C13	119.3 (4)	O3—C17A—H17A	109.00
C12—C13—C14	120.8 (4)	O3—C17A—H17B	109.00
C9—C14—C13	120.9 (4)	C18A—C17A—H17A	109.00
O2—C15A—C16A	106.0 (5)	C18A—C17A—H17B	109.00
O2—C15B—C16B	101 (2)	H17A—C17A—H17B	108.00
O3—C17A—C18A	113.3 (8)	O3—C17B—H17C	114.00
O3—C17B—C18B	87.0 (15)	C18B—C17B—H17D	114.00
C1—C2—H2	120.00	O3—C17B—H17D	114.00
C3—C2—H2	120.00	C18B—C17B—H17C	114.00
C2—C3—H3	120.00	H17C—C17B—H17D	111.00
C4—C3—H3	120.00	C17A—C18A—H18B	110.00
C3—C4—H4	120.00	C17A—C18A—H18C	109.00
C5—C4—H4	120.00	C17A—C18A—H18A	109.00
C4—C5—H5	119.00	H18A—C18A—H18C	109.00
C6—C5—H5	119.00	H18B—C18A—H18C	110.00
C1—C6—H6	120.00	H18A—C18A—H18B	109.00
C5—C6—H6	120.00	C17B—C18B—H18D	110.00
C7—C8—H8A	109.00	C17B—C18B—H18E	110.00
C7—C8—H8B	109.00	C17B—C18B—H18F	110.00
C9—C8—H8A	109.00	H18D—C18B—H18E	109.00
C9—C8—H8B	109.00	H18D—C18B—H18F	109.00
H8A—C8—H8B	108.00	H18E—C18B—H18F	109.00
C9—C10—H10	120.00		
			
O3—P1—O2—C15A	69.5 (4)	C2—C1—C7—O1	−175.6 (3)
O4—P1—O2—C15A	−54.7 (4)	C2—C1—C7—C8	−52.0 (4)
C7—P1—O2—C15A	−179.2 (4)	C6—C1—C7—P1	−106.6 (3)
O2—P1—O3—C17A	−165.6 (5)	C6—C1—C7—O1	5.5 (4)
O4—P1—O3—C17A	−40.8 (5)	C6—C1—C7—C8	129.1 (3)
C7—P1—O3—C17A	83.9 (5)	C1—C2—C3—C4	0.2 (6)
O2—P1—C7—O1	−176.6 (2)	C2—C3—C4—C5	0.0 (6)
O2—P1—C7—C1	−61.4 (3)	C3—C4—C5—C6	−0.2 (6)
O2—P1—C7—C8	64.6 (3)	C4—C5—C6—C1	0.0 (6)
O3—P1—C7—O1	−67.1 (2)	P1—C7—C8—C9	172.9 (2)
O3—P1—C7—C1	48.1 (3)	O1—C7—C8—C9	59.3 (3)
O3—P1—C7—C8	174.1 (2)	C1—C7—C8—C9	−62.4 (4)
O4—P1—C7—O1	57.9 (3)	C7—C8—C9—C10	−83.0 (5)
O4—P1—C7—C1	173.1 (3)	C7—C8—C9—C14	98.0 (5)
O4—P1—C7—C8	−60.9 (3)	C8—C9—C10—C11	−179.4 (5)
P1—O2—C15A—C16A	134.5 (4)	C14—C9—C10—C11	−0.4 (7)
P1—O3—C17A—C18A	139.4 (7)	C8—C9—C14—C13	178.7 (5)
C6—C1—C2—C3	−0.3 (5)	C10—C9—C14—C13	−0.3 (8)
C7—C1—C2—C3	−179.3 (3)	C9—C10—C11—C12	1.1 (8)
C2—C1—C6—C5	0.2 (5)	C10—C11—C12—C13	−1.1 (10)
C7—C1—C6—C5	179.2 (3)	C11—C12—C13—C14	0.4 (10)
C2—C1—C7—P1	72.4 (4)	C12—C13—C14—C9	0.3 (9)

### Hydrogen-bond geometry (Å, °)

**Table:**

*D*—H···*A*	*D*—H	H···*A*	*D*···*A*	*D*—H···*A*
O1—H1···O4^i^	0.86 (4)	1.87 (4)	2.718 (3)	170 (5)
C10—H10···O4^i^	0.93	2.54	3.409 (4)	155
C16A—H16B···CgA^ii^	0.97	2.94	3.657 (8)	133

Symmetry codes: (i) −*x*+1/2, −*y*+3/2, −*z*+1; (ii) *x*, −*y*+1, *z*+1/2.
